# The Impact of Environmental Humidity and Seasonal Changes on Blood Pressure and Major Health Complications: Myocardial Infarction, Stroke, Pulmonary Embolism, Syncope, and Mortality in Hypertensive Elderly Patients in the Eastern Mediterranean Region

**DOI:** 10.3390/life16101758

**Published:** 2026-10-20

**Authors:** Lior Charach, Lior Zusmanovitch, Ilya Novikov, Alexander Rabinovich, Gideon Charach

**Affiliations:** 1 Department of Internal Medicine C, Laniado Medical Center, Affiliated with Ariel Faculty of Medicine, Netanya 4244916, Israel; 2 Division of Gastroenterology, Laniado Medical Center, Affiliated with Ariel Faculty of Medicine, Netanya 4244916, Israel; 3 Meuhedet Health Services, Tel Aviv 62038, Israel; 4 Department of Geriatrics, Sheba Medical Center (Tel Hashomer), Affiliated with Sackler Faculty of Medicine, Tel Aviv University, 260 Sheba Rd, Ramat Gan 5262101, Israel; 5 Maccabi Health Services, Tel Aviv 6812509, Israel; 6 Faculty of Digital Technology, Holon Institute of Technology, Holon 5810201, Israel

**Keywords:** atmospheric humidity, seasonal blood pressure variations, environmental health, major complications

## Abstract

**Background**: Relative humidity is an important meteorological factor that has received considerably less attention than temperature and atmospheric pressure regarding its effects on human health. This study investigated the association between environmental humidity, blood pressure, and major cardiovascular complications in well-controlled hypertensive patients. **Methods**: In this prospective study (which was analyzed retrospectively), 128 hypertensive patients aged 65–92 years were followed for five years in a primary care clinic. Clinical evaluations were performed every season, resulting in 20 seasonal observation periods. Relative humidity (RH) was measured during each patient visit. Poisson regression was used to evaluate the associations between humidity, season, age, and the occurrence of major clinical events, including myocardial infarction, stroke, pulmonary embolism, syncope, and death. A negative Binomial model was used to check for overdispersion; no evidence of overdispersion was detected. Repeated-measures multiple linear regression analysis was performed to assess the effect of humidity on systolic and diastolic blood pressure. **Results**: Relative humidity ranged from 49% to 89% during the study period. The highest number of major clinical events occurred in winter when RH was the highest (23 events), followed by summer (10), autumn (7) and spring (3). In the unadjusted analysis, higher humidity was significantly associated with an increased number of major complications. However, this association was no longer statistically significant after adjustment for age and season. Mean systolic and diastolic blood pressure values were significantly lower in men than in women (systolic: 145.1 vs. 148.7 mmHg, *p* < 0.001; diastolic: 75.0 vs. 79.5 mmHg, *p* < 0.001). After adjustment for season, age, and body mass index, increasing humidity was associated with a significant increase in both systolic and diastolic blood pressure in men (*p* < 0.001), whereas no significant effect on systolic blood pressure was observed in women. In contrast, diastolic blood pressure decreased significantly with increasing humidity in women (*p* < 0.001). **Conclusions**: Higher relative humidity was associated with a greater number of major clinical events in the unadjusted analysis; however, this association was no longer statistically significant after adjustment for season and other relevant covariates. Seasonal variation was more strongly associated with adverse clinical outcomes than RH considered as an isolated environmental factor. Closer monitoring of blood pressure during periods of high humidity, particularly in winter, may help reduce cardiovascular risk in elderly patients with hypertension.

## 1. Introduction

The effects of environmental factors, including weather and climate, on human health have long been a subject of investigation and considerable interest [1,2,3,4,5,6,7,8]. The main components of climate and weather include temperature, seasonal variation, atmospheric pressure, and humidity.

Most environmental factors have been extensively studied, and their effects on human health have been demonstrated. Blood pressure is considered one of the most widely investigated clinical parameters because of its close relationship with major cardiovascular risk factors, particularly in patients with hypertension (HTN). Variations in atmospheric pressure, temperature, and season may affect BP [1,2,3,8].

Relative humidity is an important environmental parameter that may substantially affect human health. An RH range of approximately 40–60% profoundly impacts health. High humidity (>60%) may impair sweat evaporation and heat dissipation, potentially contributing to overheating, dehydration, and increased cardiovascular strain. High humidity also promotes the growth of mold and dust mites, which may exacerbate asthma and allergic disorders. Conversely, low humidity (<30%) may cause dryness of the skin, eyes, and mucous membranes and may increase susceptibility to respiratory infections [9,10,11,12].

Relative humidity remains an underestimated parameter of both indoor and outdoor environments. Conditions below or above the optimal RH range may facilitate the transmission of infectious agents and exacerbate respiratory diseases and allergic diseases. Although RH has historically received less attention than other environmental factors, such as air pollution and temperature, recent studies have emphasized its potential health effects, especially in the elderly population [9,10,11,12,13,14].

Previous studies evaluating short-term dehumidification have suggested that reducing the RH in humid indoor environments may improve clinical and biochemical measures of cardiorespiratory health. For example, an RH of 46.9 ± 8.7% in dehumidified rooms was suggested to be more favorable than an RH of 75.2 ± 7.9% in humid rooms [15]. Humidity also contributes to the development of atherosclerosis through oxidative stress and the release of inflammatory mediators. In addition, oxidative stress-induced platelet activation may promote thrombosis and thereby increase cardiovascular risk in older adults [9,10,13,15].

Most previous studies have not demonstrated a strong independent association between humidity and cardiovascular complications [16,17,18,19].

Meteorological conditions and their relationships with atmospheric pressure differ among geographic regions. In temperate and continental climates, atmospheric pressure may vary substantially with temperature and season. In the Eastern Mediterranean region, winter climatic conditions are relatively stable, and atmospheric pressure is generally high [7,8,18,19,20].

Numerous studies have investigated the association between cold weather and the incidence of cardiovascular complications, including myocardial infarction, stroke, and aortic dissection [6,9,15,16,17,18]. Weather conditions may exert important effects on the autonomic nervous system. Catecholamine concentrations increase during exposure to cold weather, resulting in activation of the sympathetic nervous system [21,22,23]. Associations among atmospheric pressure, BP, and temperature changes have also been reported [18,19,20,21,22]. Blood pressure tends to be lower in warmer and tropical climates [23,24]. An inverse association between atmospheric pressure and BP during spring has also been observed [3]. In Israel, higher atmospheric pressure during winter and lower atmospheric pressure during summer are common meteorological patterns [7,8,20].

Hypertension is a well-established risk factor for cardiovascular and cerebrovascular events. The incidence of these complications is higher during winter than during summer, particularly among older adults [20,23,24]. However, considerably less is known about the effect of humidity as an independent meteorological factor on mortality and major cardiovascular complications.

Previously, we reported greater seasonal BP variation among older patients with hypertension, with mean systolic blood pressure (SBP) and diastolic blood pressure (DBP) values being higher during winter than during summer [6]. The winter–summer difference exceeded 30 mmHg for SBP and 14 mmHg for DBP. A higher frequency of fatal events among older individuals has also been reported [6,13,14,15].

The independent contribution of RH to BP variation and major cardiovascular complications remains unclear. Because humidity frequently varies together with other meteorological factors, particularly temperature, season, and atmospheric pressure, its specific effect on cardiovascular outcomes is difficult to determine.

The aim of this prospective study was to evaluate the association between environmental RH and BP in older patients receiving treatment for hypertension and to investigate its relationship with major clinical outcomes, including myocardial infarction, stroke, pulmonary embolism, syncope, and mortality, during a 5-year follow-up period.

## 2. Methods

### 2.1. Patients

The patients recruited for this prospective study had a diagnosis of essential hypertension, and the data were subsequently analyzed retrospectively. They were treated and followed in a Maccabi Health System primary care outpatient clinic from January 2008 to December 2012. The study was approved by the institutional ethics committee of Maccabi Health Services (IRB number: MHS-0173-07). The patients gave their consent for participation in place at enrollment.

Patients with elevated BP (SBP 141–200 mmHg and DBP 90–115 mmHg) and aged ≥65 years were recruited for the study. Exclusion criteria included: previous stroke, recent myocardial infarction, acute coronary syndrome, congestive heart failure, advanced oncologic disease (patients with metastases receiving oncological treatment chemotherapy or radiotherapy), chronic obstructive pulmonary disease (dyspnea and pulmonary function test FEV1/FVC ratio of less than 0.70 and diagnosis of emphysema (chest X-ray) or chronic bronchitis), moderate and severe pulmonary hypertension (mean pulmonary artery pressure > 55 mmHg), chronic kidney disease (creatinine > 1.4 mg/dL and GFR < 60, previous criteria), anemia (hemoglobin < 12 g%), secondary HTN, hyperthyroidism or hypothyroidism, chronic inflammatory diseases, cognitive disorders and advanced dementia (profound cognitive failure and loss of physical independence and MMSE < 10), and hypercoagulability syndromes (most of which are risk factors for stroke, myocardial infarction, pulmonary emboli, syncope, and mortality).

Antihypertensive treatment included ACE inhibitors, angiotensin receptor blockers, calcium channel blockers, diuretics, β-blockers, or α-blockers. The patients continued their regular daily activities and continued consuming their usual amounts of caffeine, tobacco, and alcohol.

### 2.2. Study Protocol

Relative humidity in the clinic was measured at each patient visit and compared with humidity data from the Israeli Meteorological Service. Humidity was measured in the clinic and was similar to that in patients’ home environments according to the responses (patients questioning) obtained during the visit. The humidity measurements were similar to data from the Israeli Meteorological Service.

Patients’ current and past medical histories were recorded, and a physical examination was performed by the same physician for all patients, including weight measurements. All patients underwent electrocardiography, chest X-rays, retinal examinations, and echocardiography during the observation period. Blood tests included analyses of renal function, electrolyte levels, lipid profile, and complete blood counts. Blood pressure was measured after a 15 min rest, which is acceptable for adaptation to the environmental conditions of temperature and humidity in a physician’s clinic, as appropriate to the Middle Eastern climate, where very low temperatures (<0 °C) and high temperatures (>35 °C) are rare (Israel Meteorological Service); thus, adjustment is quite quick. BP was measured using a mercury sphygmomanometer with a standard-sized cuff (12 cm × 35 cm). For patients with BMI > 27 kg/m^2^ and/or with a large upper arm, a 16 × 36 cm cuff was used. For patients with an upper arm circumference of 45–52 cm, a 16 × 42 cm (extra-large) cuff was used. Blood pressure was considered normal when SBP was ≤140 mmHg and DBP was ≤90 mmHg. All BP examinations were performed in the same physician’s room by the same physician (G.C.).

During the 5-year period, the patients were followed up at clinically indicated intervals, which did not exceed 3 months, under the same room temperature conditions. Humidity and BP were recorded at each visit. The atmospheric relative humidity, expressed as a percentage, was compared with humidity data from the Israeli Meteorological Service (Beit Dagan, Israel Meteorological Service). Mean year-round humidity values are presented in the figure. However, to associate blood pressure with atmospheric pressure (AP), readings on the visit days were specifically recorded for data analysis.

Weight was measured and body mass index (BMI) was calculated at the first visit and was reassessed every 6 months.

Antihypertensive treatment included diuretics in 62% of patients (hydrochlorothiazide—38%, furosemide—22%, and spironolactone—14%); beta-blockers in 54%; calcium channel blockers in 49%; angiotensin-converting enzyme inhibitors/angiotensin receptor blockers in 74%; and alpha blockers in 10%. Additional antihypertensive treatment was required under high-humidity conditions and the cold season in 26% of patients.

Among the patients, 14 had diabetes mellitus, and 32 were smokers.

## 3. Statistical Analysis

The data are presented overall and by season using standard descriptive statistics: the number of observations (count), mean, standard deviation, median, minimum, and maximum. The scales of continuous variables were changed for easier interpretation: the minimum value of 65 was subtracted from age, and humidity and BMI were centered by subtracting their median values (69 and 26). We defined the categorical subgroups of patients as aged ≥75 versus <75 and BMI ≥ 26 versus <26. The effect of humidity on the number of clinical events was analyzed by performing simple and multiple Poisson regression on humidity only and with adjustment for season and age. Because of the limited number of complications and the mixture of different types of events, we avoided potential relationships between recurrent episodes and assumed independence. A negative Binomial model was used to assess overdispersion, and no evidence of overdispersion was detected. The effect of humidity on BP was analyzed using a repeated-measures model with humidity, season, gender, age, and BMI at inclusion as fixed effects and patient as a random effect. We assumed equal within-patient correlation between the measurements of BP because of the strong variability of BP and the long (three months) intervals between the consecutive measurements. We tested pairwise interactions between humidity and gender and the subgroups of older versus younger and higher versus lower BMI. All calculations were performed using STATA 14 software. No corrections for multiple comparisons were performed.

## 4. Results

A total of 142 patients with hypertension were initially enrolled according to the predefined inclusion and exclusion criteria. Fourteen patients were excluded because of poor compliance, insufficient follow-up data, active ischemic heart disease, congestive heart failure, previous stroke, previous pulmonary embolism, recurrent syncope, or death occurring within the first 3 months of the study.

The remaining 128 patients, comprising 60 men and 68 women, were followed for 5 years. The mean age of the study population was 75.7 (range, 65–92 years), and the mean body mass index (BMI) was 26.9 ± 5.4 kg/m^2^. BMI did not change significantly during the follow-up period and did not affect BP.

The main demographic and clinical characteristics of the study population are presented in Table 1. The mean humidity during the 5-year study period was 68.7 ± 6.9. The mean systolic blood pressure (SBP) was 146.8 ± 17.96 mmHg, and the mean diastolic blood pressure (DBP) was 77.1 ± 11.7 mmHg. The majority of the patients were not obese, and their BP was within the target range.

The dataset for the analysis of clinical events consisted of 20 seasonal observations, with each row representing a unique year-by-season combination. The variables included the seasonal average humidity and counts of myocardial infarction, stroke, pulmonary embolism, syncope, and mortality, as well as the composite event count variable, defined as the sum of all five event categories.

A total of 18 patients (14%) developed 43 major complications during the 5-year observation period: MI was recorded in 11 patients, stroke in 6, PE in 6, and syncope (noncardiac) in 14. Seven patients died during this period.

Table 2 presents the absolute numbers of clinical events and average RH according to season and year. The total numbers of events during the five-year observation period were as follows: MI, 11; CVA, 6; PE, 6; syncope, 14; and death, 7. Remarkably, the majority of clinical events occurred in cold seasons.

The overall events recorded over the five-year study period are summarized in Table 3.

Humidity showed relatively limited variation over the study period, with a mean of 68.7, median of 69, standard deviation of 6.7, and a range of 49–89. The event variables were sparse and right-skewed, with many seasonal observations containing zero events. The total number of events per season had a mean of 2.2, a median of 2, an interquartile range of 1 to 3, and a maximum of 9.

**Figure 1:**
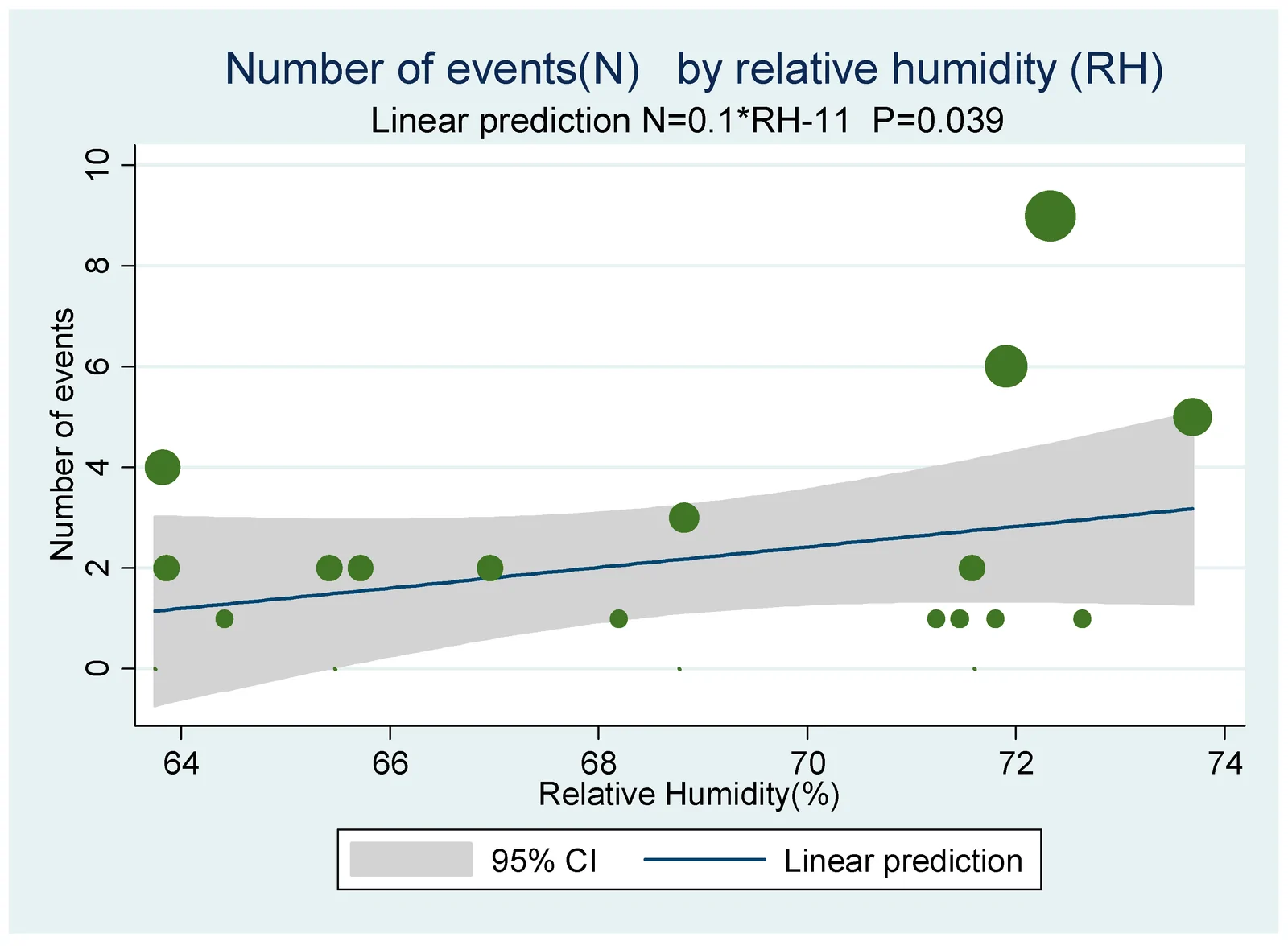
Number of clinical events versus average relative humidity in the season.

The relationship between humidity and the number of events is shown in Figure 1, which presents the number of events by season versus the average humidity of the season. The area of the circles is proportional to the number of events. The total number of events (n = 43) is also illustrated in Figure 1.

A multiple Poisson regression model was fitted with the number of events as the dependent variable and year, humidity, and season as the predictors, with winter as the reference season (Table 4). A negative Binomial model was used to assess for overdispersion, and no evidence of overdispersion was detected. The model demonstrates a significant positive association with year, with an incidence rate ratio of 1.30 and a 95% confidence interval of 1.03 to 1.62, indicating that the expected seasonal event count increased by approximately 30 percent per year after adjustment for humidity and season. Humidity was inversely associated with event count, but this association was not statistically significant, with an incidence rate ratio of 0.874 and a 95% confidence interval of 0.65 to 1.175. Compared with winter, autumn was associated with a significantly lower expected event count, with an incidence rate ratio of 0.201 and a 95% confidence interval of 0.068 to 0.596, and spring had significantly fewer complications, with an incidence rate ratio of 0.076 and a 95% confidence interval of 0.009 to 0.651; however, the effect of summer did not differ significantly from that of winter.

We did not investigate the potential interactions between humidity, season, and year because of the small total number (n = 43) of events.

These findings suggest that event burden increased over time and was highest during winter, while humidity did not show a statistically clear association with the total seasonal event count. Given the small number of observations, these results should be interpreted as exploratory. The apparent discrepancy between the univariable and multivariable analyses likely reflects confounding. Humidity varies strongly between seasons. A one-way ANOVA across the four seasonal groups yielded F (3, 2556) = 224.75, *p* < 0.0001. This explains why the significant positive effect of humidity alone became non-significant (weakly negative) in the multiple regression.

**Figure 2:**
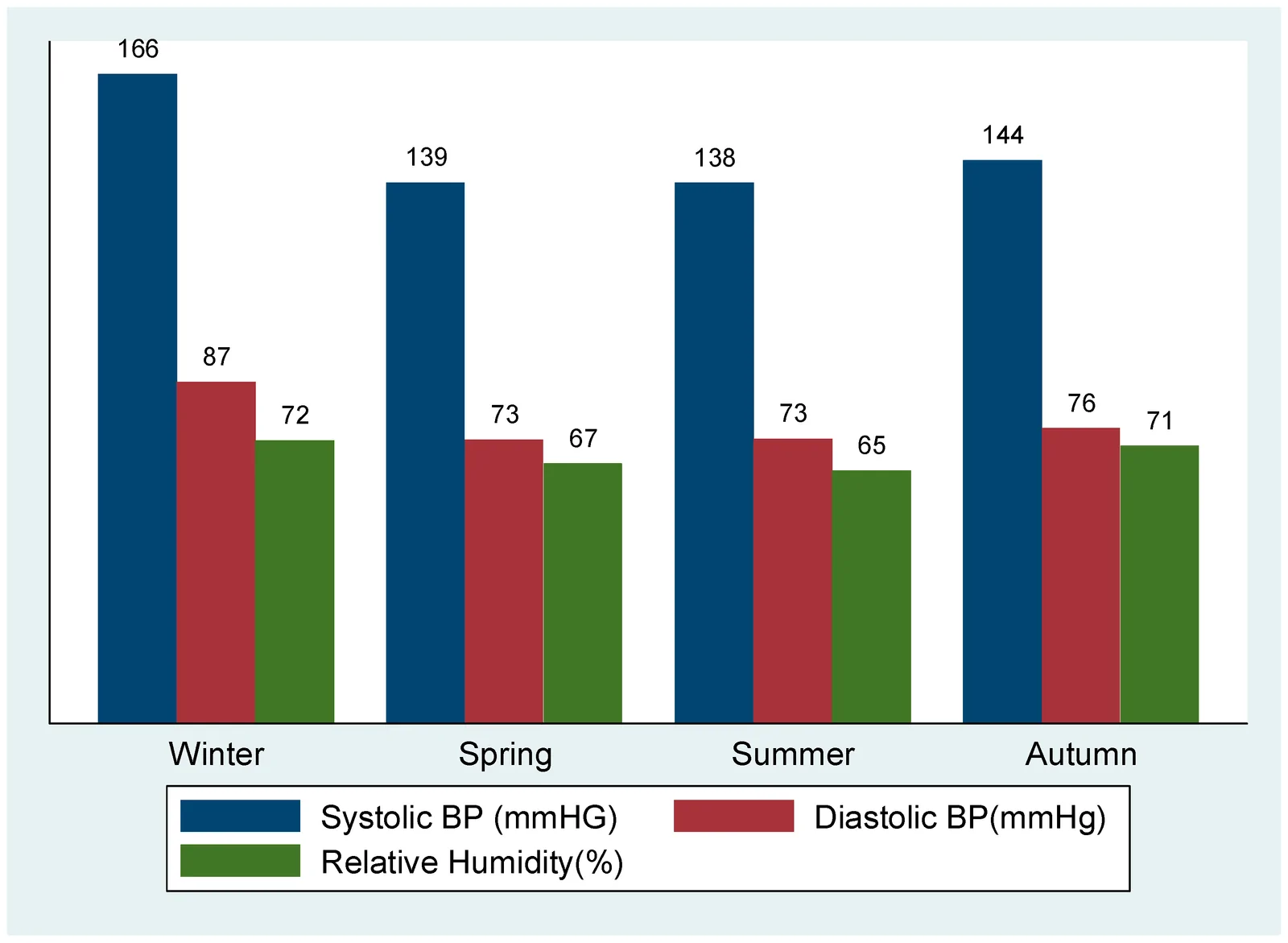
Mean humidity values and systolic and diastolic blood pressure according to season.

**Figure 3:**
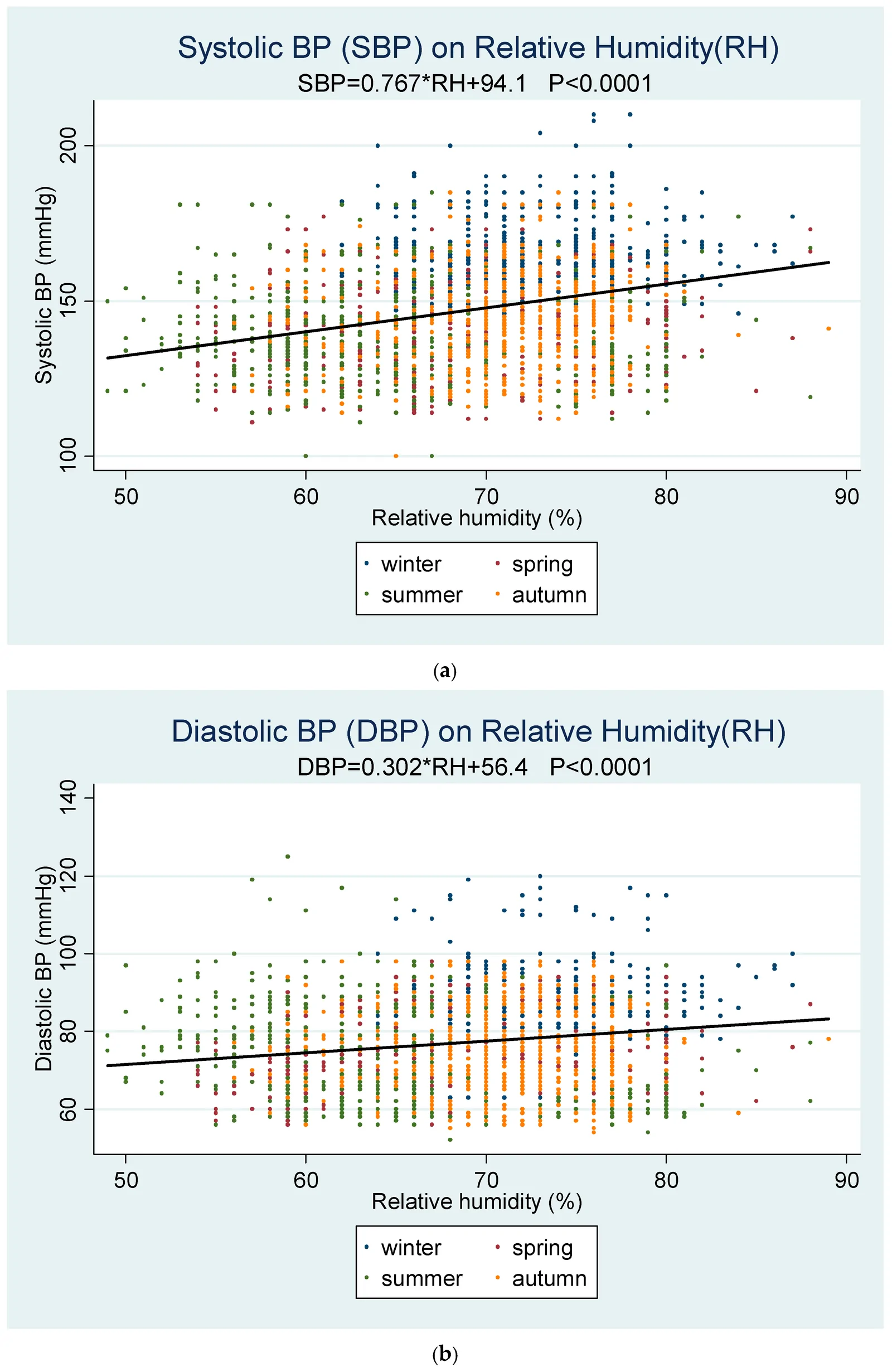
(**a**) Association of systolic blood pressure with humidity and seasons. (**b**) Association of diastolic blood pressure with humidity and seasons.

The relationships among systolic BP, diastolic BP, humidity, and season are presented in Table 5 and Figure 2 and Figure 3.

After adjustment for season, age, and BMI, the estimated effect of humidity on systolic blood pressure (Table 6) in men there was an increase of 0.341 mmHg per one percentage point in RH (*p* < 0.001), corresponding to an approximately 14 mmHg difference across the observed RH range of 49–89%. No significant effect was observed in women (*p* = 0.99). For diastolic blood pressure, humidity was associated with a significant decrease of 0.252 mmHg per unit increase in women (*p* < 0.001), while in men, it was associated with a significant increase of 0.166 mmHg per unit increase (*p* < 0.001). Seasonal terms remained strongly associated with both outcomes (SBP and DBP), indicating substantially lower blood pressure levels outside the winter season.

Explanation of the model. The reference value represents the estimated mean outcome (systolic or diastolic blood pressure) during the winter of the first year of observation in women aged 65 years with BMI = 26 and humidity = 69. The effect of season (spring, summer, and autumn) represents the difference between the estimated mean outcome value in that season and the corresponding value in winter. The coefficient for male represents the estimated difference in outcome between men and women. The coefficients for age (65), BMI (26), and humidity (69) represent the estimated changes in the outcome associated with a one-unit increase in age, BMI, and humidity, respectively. The interaction term male × humidity represents the difference between the effects of a one-unit increase in humidity in men versus women. Additional analyses of potential pairwise interactions between humidity and season, age subgroup (<75 vs. ≥75 years), and BMI subgroup (<26 vs. ≥26) did not reveal any statistically significant interactions. This indicates that the effect of humidity did not differ significantly across these subgroups. The model showed that the estimated mean diastolic value in the reference category was 89.73 mmHg, and the values were, on average, 14.21 mmHg lower in spring, 15.57 mmHg lower in summer, 12.07 mmHg lower in autumn, and 4.31 mmHg lower in men. Changes with age and BMI did not significantly differ from zero. A one percentage point increase in relative humidity decreased DBP in women by 0.252 mmHg, whereas it increased DBP in men by 0.166 mmHg. The systolic BP value in the reference category was 167.53 mmHg, and the values were on average 26.06 mmHg lower in spring, 27.11 mmHg lower in summer, 22.01 mmHg lower in autumn, and 3.72 mm Hg lower in males. Changes with age and BMI did not significantly differ from zero. A one percentage point increase in relative humidity was associated with a 0.342 mmHg increase in SBP, whereas it had no significant effect in women. Additional antihypertensive treatment was required during high humidity (72%) in 26% of selected patients.

## 5. Discussion

This prospective study evaluated the association between environmental relative humidity, systolic blood pressure, diastolic blood pressure, and major clinical events in older patients with treated hypertension during a 5-year follow-up period. The principal findings were that BP varied substantially according to season and that the association between RH and BP differed significantly between men and women. In addition, the highest number of major clinical events occurred during winter. High RH was significantly associated with the total number of clinical events and higher BP in unadjusted analysis.

However, this association was no longer statistically significant after adjustment for season and year of follow-up. These findings suggest that seasonal variation may have a stronger influence on cardiovascular risk than RH considered as an isolated meteorological factor. Higher RH was associated with increased BP. Although RH was also associated with a greater number of major cardiovascular complications in the unadjusted analysis, this association was not maintained after multivariable adjustment.

The seasonal variation in BP observed in the present study is consistent with previous reports demonstrating higher BP during colder periods [6,20]. In our study, both systolic blood pressure and diastolic blood pressure were highest during winter and substantially lower during spring, summer, and autumn. After adjustment for RH, sex, age, and body mass index, season remained strongly associated with both SBP and DBP. Compared with winter, SBP was approximately 22–27 mmHg lower during the other seasons, whereas DBP was approximately 12–16 mmHg lower. These findings support the concept that seasonal and environmental factors may have clinically important effects on BP regulation in older patients with hypertension. In the present study, temperature was not analyzed. Instead, we examined RH and season, both of which are closely related to temperature. Given the advanced age of the participants, this approach took into account their thermal comfort and concerns about excessive cooling. Consequently, indoor environmental conditions were not consistently controlled, and participants were likely exposed to variations in ambient humidity during a substantial portion of the daytime. In the Eastern Mediterranean region, winter is generally characterized by lower temperatures than the other seasons.

Several physiological mechanisms may explain the association between cold weather and elevated BP. Exposure to cold activates the sympathetic nervous system and increases catecholamine concentrations, resulting in peripheral vasoconstriction and increased vascular resistance [21,22,23]. Cold exposure may also increase renal sympathetic nerve activity and contribute to sustained elevations in BP [22]. These physiological responses may be particularly relevant in patients with hypertension, in whom vascular and autonomic regulatory mechanisms may already be impaired.

Previous studies have also demonstrated associations among atmospheric pressure, temperature, seasonal changes, and BP [3,18,19,20,21,22]. In our previous investigation, substantial winter–summer differences in SBP and DBP were observed among older hypertensive patients [6]. The present findings are consistent with these observations and further emphasize the importance of seasonal variation in BP control. In addition, the increased number of major clinical events observed during winter is consistent with reports of increased cardiovascular and cerebrovascular morbidity during colder periods [20,23,24].

An important finding of the present study is the significant interaction between RH and sex in relation to BP. After adjustment for season, age, and BMI, increasing RH was associated with higher SBP and DBP in men. In women, RH had no significant effect on SBP and was inversely associated with DBP. These findings suggest that the physiological response to environmental humidity may differ between men and women.

The mechanisms underlying these sex-specific differences remain uncertain. Differences in autonomic regulation, vascular reactivity, hormonal factors, body composition, and thermoregulatory responses may contribute to different cardiovascular responses to environmental conditions. However, because the present study was not specifically designed to investigate the biological mechanisms underlying sex-related differences, these findings should be considered exploratory and require confirmation in larger studies.

The relationship between RH and cardiovascular disease remains controversial. Previous studies have not consistently demonstrated an independent association between humidity and cardiovascular complications [3,9,15,16,17,18,19]. Some studies have suggested that humidity may influence cardiovascular health through oxidative stress, inflammation, endothelial dysfunction, and platelet activation [9,10,13,15,22]. Oxidative stress may promote inflammatory mediator release and contribute to the development of atherosclerosis. Furthermore, oxidative stress-induced platelet activation may increase thrombotic risk, particularly among older individuals [9,10,13,15,22].

In the present study, RH was positively associated with the total number of major clinical events in the unadjusted analysis. However, this association was not statistically significant after adjustment for season and year of follow-up. Therefore, our findings do not establish RH as an independent risk factor for myocardial infarction, stroke, pulmonary embolism, syncope, or mortality. Instead, the observed unadjusted relationship between RH and clinical events may partly reflect the close relationship between humidity and broader seasonal meteorological conditions.

Meteorological factors do not act independently under real-world conditions. Temperature, atmospheric pressure, humidity, wind, and other weather-related factors change simultaneously and may interact with one another [5,6,7,8,15,18,20,21,22,23]. This may explain why season was more strongly associated with clinical events than RH alone in the present study. Season may represent the combined effects of several environmental exposures and physiological adaptations rather than the effect of a single meteorological parameter.

The year of follow-up was also significantly associated with the total number of clinical events, with an approximately 30% increase in the expected seasonal event count per year after adjustment for RH and season. This finding may reflect the increasing age and cumulative cardiovascular risk of the study population during the 5-year follow-up period. However, because of the relatively small number of clinical events, this observation should be interpreted cautiously.

Hypertension is a well-established major risk factor for cardiovascular and cerebrovascular diseases. Consequently, environmentally associated fluctuations in BP may potentially contribute to variations in cardiovascular risk. In the present study, the marked seasonal differences in BP occurred despite antihypertensive treatment. Moreover, additional antihypertensive treatment was required in a proportion of patients during periods of high RH. These observations suggest that environmental conditions, including RH, may have clinical relevance for BP management in older hypertensive patients.

Nevertheless, the present findings should not be interpreted as evidence that high RH directly causes cardiovascular complications. The adjusted analysis did not demonstrate an independent association between RH and the total number of clinical events. Rather, our findings suggest that RH may contribute to BP variation in a sex-specific manner, whereas broader seasonal conditions, including temperature and atmospheric pressure, appear to be more strongly associated with major clinical outcomes.

These findings may have practical clinical implications. Older patients with hypertension may benefit from closer BP monitoring during periods of substantial seasonal and meteorological changes, particularly during winter. Awareness of environmentally associated BP variation may help physicians identify patients who require closer follow-up or adjustment of antihypertensive therapy. However, treatment decisions should remain individualized and based on repeated BP measurements rather than meteorological conditions alone.

Further prospective studies involving larger and more geographically diverse populations are required to clarify the independent effects of RH on BP and cardiovascular outcomes. Future investigations should simultaneously assess RH, temperature, atmospheric pressure, wind speed, and other environmental variables and should evaluate potential interactions among these factors. Larger sample sizes would also allow separate analyses of individual cardiovascular outcomes and more detailed investigation of sex-specific responses to environmental humidity.

### Limitations

The present study has several limitations. First, the relatively small sample size and limited number of clinical events reduced the statistical power of the analysis and prevented separate analyses of individual cardiovascular outcomes. The analysis assumed independence among the 43 events occurring in 18 patients (mean, 2.4 events per affected patient). Several potential cardiovascular risk factors were not included in the statistical models, and residual confounding cannot be excluded. In addition, meteorological parameters such as temperature, atmospheric pressure, and wind speed were not simultaneously assessed. Because these environmental factors may interact with RH and vary according to season, the independent effect of relative humidity on clinical events (PE, myocardial infarction, stroke, syncope, and death) is difficult to determine. Finally, this was a single-center observational study of older patients with treated hypertension in the Eastern Mediterranean region. Therefore, the findings may not be generalizable to other populations or climatic regions, and causal relationships cannot be established. Larger prospective studies incorporating multiple meteorological variables are needed to confirm these findings.

## 6. Conclusions

The present study demonstrates that higher RH was associated with increased BP. Although RH was also associated with a greater number of major cardiovascular complications in the unadjusted analysis, this association was not maintained after multivariable adjustment.

The study found substantial seasonal variation in BP among older patients with treated hypertension. Both SBP and DBP were highest during winter, and season remained strongly associated with BP after adjustment for RH, sex, age, and BMI.

The association between RH and BP differed significantly according to sex. Increasing RH was associated with higher SBP and DBP in men, whereas no significant association with SBP and an inverse association with DBP were observed in women. These findings suggest that cardiovascular responses to environmental humidity may differ between men and women. The highest number of major clinical events occurred during winter. Although increasing RH was associated with a greater number of clinical events in the unadjusted analysis, RH was not independently associated with event burden after adjustment for season and year of follow-up. Therefore, the present findings do not establish RH as an independent risk factor for major cardiovascular complications or mortality.

Overall, seasonal variation appears to have a stronger association with BP and major clinical outcomes than RH considered as an isolated environmental factor. Closer BP monitoring during periods of substantial seasonal and meteorological changes, particularly during winter, may be appropriate in older patients with hypertension. Further large-scale prospective studies are needed to clarify the independent contribution of RH and its interaction with other meteorological factors to BP regulation and cardiovascular risk.

## Figures and Tables

**Table 1:** Main demographic and clinical patient data, which include age, BMI, humidity, and systolic and diastolic blood pressure.

Variable	Number	Mean	SD	Median	Min	Max
Age at enrollment	128 patients	75.0 years	6.44	74.5	65	92
BMI at enrollment	128 patients	26.9	5.42	26	15	41
Relative humidity	2560 measurements	68.7%	6.86	69	49	89
Systolic BP	2512 measurements	146.8 mmHg	17.76	145	100	210
Diastolic BP	2510 measurements	77.1 mmHg	11.69	77	52	125

**Table 2:** Absolute number of clinical events according to year, season, and mean humidity.

Year	Season	MI	Stroke	PE	Syncope	Death	Events	Humidity
2008	Autumn	1	0	0	0	0	1	71.24
2008	Spring	0	0	0	0	0	0	68.19
2008	Summer	0	0	0	2	0	2	66.96
2008	Winter	1	0	1	0	0	2	71.57
2009	Autumn	1	0	0	0	0	1	71.46
2009	Spring	0	0	0	0	0	0	65.47
2009	Summer	0	0	0	2	0	2	65.42
2009	Winter	0	0	1	0	0	1	72.09
2010	Autumn	1	0	0	0	0	1	71.60
2010	Spring	0	1	0	0	0	1	64.42
2010	Summer	0	0	0	0	0	0	63.75
2010	Winter	1	1	2	1	1	6	71.37
2011	Autumn	0	0	0	0	1	1	71.80
2011	Spring	1	0	0	1	0	2	65.72
2011	Summer	1	1	0	2	0	4	63.82
2011	Winter	2	2	1	2	2	9	71.77
2012	Autumn	0	1	0	2	0	3	68.82
2012	Spring	0	0	0	0	0	0	68.77
2012	Summer	0	0	1	1	1	2	63.85
2012	Winter	1	1	0	1	2	5	72.04

All syncope events (including postural hypotension) in this study were noncardiac, which may explain why they were more common during hot periods (sweating and dehydration).

**Table 3:** Number of events per season.

Season	MI	Stroke	PE	Syncope	Death	Events (n)
Winter	5	4	5	4	5	23
Spring	1	0	0	1	0	2
Summer	1	1	1	7	1	11
Autumn	3	1	0	2	1	7

**Table 4:** Poisson regression of the total number of events on relative humidity, season, and year.

Factor *	IRR	*p*-Value	95% CI	
Humidity %	0.874	0.375	0.65	1.175
Spring	0.076	0.019	0.009	0.651
Summer	0.144	0.139	0.011	1.870
Autumn	0.201	0.004	0.068	0.596
Year of follow-up	1.298	0.023	1.036	1.625
Constant	0.110	0.248	0.002	4.628

* The offset was stated as logarithm of person-time.

**Table 5:** Season-specific data on systolic and diastolic blood pressure and relative humidity.

Season	Humidity (%)			Systolic BP (mmHg)			Diastolic BP (mmHg)		
	N *	Mean	SD	N *	Mean	SD	N *	Mean	SD
Winter	640	72.4	4.45	624	166.3	11.6	623	87.5	9.11
Spring	640	66.5	6.87	630	138.5	13.75	629	72.65	8.45
Summer	640	64.76	7.59	628	138.43	14.32	628	72.92	12.17
Autumn	640	70.98	4.94	630	144.10	14.81	630	75.52	9.88

* Number of measurements. Systolic BP, diastolic BP, and RH were highest in winter, followed by autumn.

**Table 6:** Repeated-measures multiple linear model.

Factor	Diastolic	Systolic
	Effect *, (Standard Error), *p*-Value	Effect *, (Standard Error), *p*-Value
Spring	−14.21, (0.581), <0.001	−26.06, (0.82), <0.001
Summer	−15.57, (0.608), <0.001	−27.11, (0.85), <0.001
Autumn	−12.07, (0.542) <0.001	−22.01, (0.76), <0.001
Male	−4.31, (0.400), <0.001	−3.72, (0.54), <0.001
Age-65	−0.021, (0.031), 0.499	0.012, (0.042), 0.771
BMI-26	0.013 (0.036), 0.717	0.004, (0.049), 0.941
Humidity-69	−0.252, (0.045) <0.001	0.0001, (0.064), 0.999
Male humidity	0.418, (0.061), <0.001	0.342, (0.085), <0.001
Reference	89.73, (1.12), <0.001	167.53, (0.743), <0.001

* Effect is the change in outcome with a one-unit change in a factor.

## Data Availability

The datasets generated and/or analyzed in this study are not publicly available due to institutional and confidentiality restrictions, but they may be obtained from the corresponding author upon reasonable request.
